# High conversion rate to total hip arthroplasty after hemiarthroplasty in young patients with a minimum 10 years follow‐up

**DOI:** 10.1186/s12891-021-04153-4

**Published:** 2021-03-12

**Authors:** Nam Hoon Moon, Won Chul Shin, Min Uk Do, Sang Woo Kang, Sang-Min Lee, Kuen Tak Suh

**Affiliations:** 1 Department of Orthopaedic Surgery, Pusan National University Hospital, Busan, Republic of Korea; 2 Department of Orthopaedic Surgery, Pusan National University Yangsan Hospital, Pusan National University School of Medicine, Research Institute for Convergence of Biomedical Science and Technology, 20 Geumo-ro, Mulgeum-eup, Gyeongsangnam-do 626-770 Yangsan, Republic of Korea

**Keywords:** Hemiarthroplasty, Young patient, Total hip arthroplasty, Acetabular erosion

## Abstract

**Background:**

This study aimed to evaluate the follow-up results of bipolar hemiarthroplasty (BHA) for more than 10 years in patients aged < 60 years and to analyze the risk factors for acetabular erosion after BHA.

**Methods:**

This retrospective study included 114 patients who underwent BHA were followed-up for at least 10 years. The mean age was 54.1 years, and the mean follow-up duration was 13.8 years. The patients were divided into two groups according to the presence of acetabular erosion, and the preoperative parameters were compared between the two groups. Moreover, the risk factors related to acetabular erosion after BHA were analyzed using statistical comparisons.

**Results:**

Reoperation was performed in 44 of the 114 patients (38.6 %). The survival rate when the end point was reoperation related to acetabular erosion was found to be significantly time-dependent: 73.2 % at 5 years, 48.8 % at 10 years, and 25.9 % at 15 years. The acetabular erosion group showed significantly younger age at the time of surgery, higher body mass index (BMI), more avascular necrosis of the femoral head, and smaller prosthetic femoral head. The final multivariate logistic regression analysis showed that young age at the time of surgery were independent risk factors for acetabular erosion after BHA in patients aged < 60 years.

**Conclusions:**

The minimum 10-year follow-up outcomes of BHA in patients aged < 60 years showed a relatively high conversion rate to total hip arthroplasty. When considering BHA in younger patients, more careful decisions should be made with respect to patient’s choice, keeping in mind that long-term survival cannot be guaranteed.

## Background

Hip arthroplasty is one of the preferred methods for ensuring success in the treatment of end-stage hip disease and trauma. Although the usage has gradually decreased owing to recent developments in implants for total hip arthroplasty (THA) and the dramatic reduction in the wear rate of the liner, bipolar hemiarthroplasty (BHA) still has some advantages, including reduced dislocation rate after surgery, shorter surgical time, less blood loss, and lower initial cost because only the femoral side is replaced [1–3]. Moreover, in the past, when the technique of THA was less developed, BHA was widely used for the treatment of various hip diseases and trauma. In the early 1980 s, some centers expanded the indications to include the primary treatment of degenerative arthritis of the hip [4]. Although internal fixation would be the first alternative for femoral neck fracture in young patients, high reoperation rate after internal fixation and better patient-reported outcome after both THA and BHA with medium follow-up have been reported [5, 6]. In proximal femur fractures such as femoral neck fractures with an intact acetabular cartilage, BHA remains an option that can be expected to be successful [7]. Particularly, it is still limitedly used as a salvage procedure in elderly patients with low functional requirements [8, 9].

However, as BHA is mostly used in patients with advanced age or poor medical condition, collection of long-term follow-up data is highly difficult. Consequently, long-term results are very rarely reported. Further, it is known that the difference in hardness between the acetabulum and the prosthetic femoral head gradually leads to loss of the acetabular cartilage and erosion and destruction of the acetabulum, such as in arthritis, over time [10, 11]. However, whether these results are due to the patients’ age or activity, poor bone quality, other underlying diseases, or implant differences is still undetermined.

Therefore, this study aimed to evaluate the follow-up results of BHA for more than 10 years in relatively young patients, focusing on the erosion and destruction of the acetabular side. Further, we intended to analyze the risk factors for acetabular erosion and destruction after BHA. We hypothesized that BHA cannot guarantee long-term results in young patients, particularly in terms of damage to the acetabulum.

## Materials and Methods

**Fig. 1:**
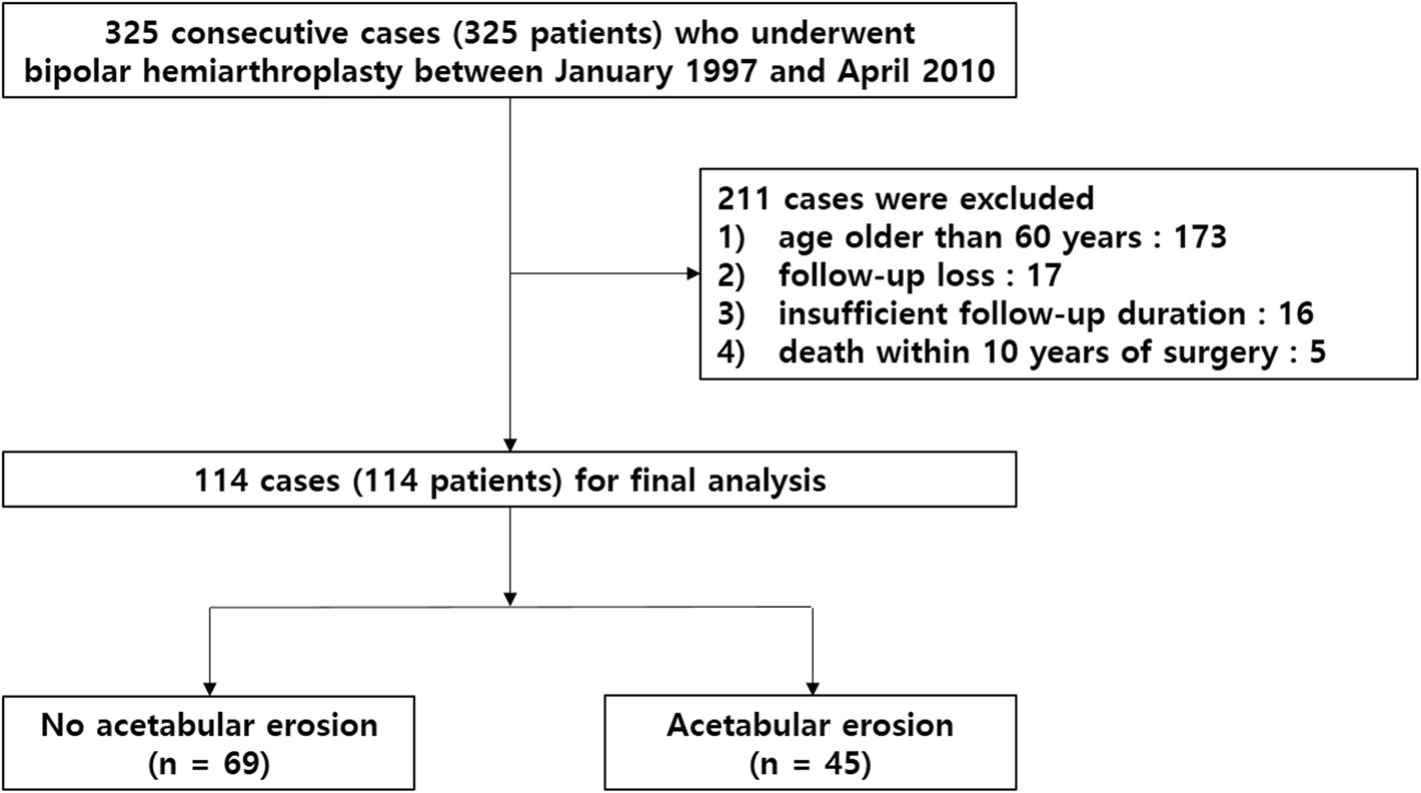
Study design flowchart

This study followed the World Medical Association Declaration of Helsinki and strengthening the reporting of observational studies in epidemiology (STROBE) guidelines for cohort studies. Patient information was reviewed by the university human subjects committee and informed consent exemption was obtained from the IRB of our affiliated institutions (Pusan National University Yangsan Hospital, Approval No. 05-2020-178). This single-center retrospective comparative cohort study enrolled patients who underwent BHA with implants from a single manufacturer. From January 1997 to April 2010, 325 BHA procedures were conducted at our tertiary university hospital. Patients prior to 1997 were not included due to the preservation of medical records and the time of a full-fledged start of hip arthroplasty. The inclusion criteria were: age < 60 years, BHA surgery, and > 10 years follow-up. Of the 325 patients, 173 patients aged > 60 years and 17 patients who were lost to follow-up, despite extensive efforts to contact them to return for radiologic evaluation, were excluded. Sixteen patients were further excluded because of insufficient long-term follow-up duration. Five patients died within 10 years of surgery. After the exclusion, 114 patients (follow-up rate, 75 %) with a minimum follow-up of 10 years were finally included (Fig. 1). The mean age at the time of surgery was 54.1 years (range, 29–59 years), and the patients comprised 51 man and 63 women. The mean follow-up period was 13.8 years (range, 10–23 years), preoperative body mass index (BMI) was 22.0 kg/m^2^ (range, 16.0-32.8 kg/m^2^). Bone mineral density (BMD) was done preoperatively except 5 cases in which preoperative examination was not performed. T-score of BMD at an unaffected trochanteric region or lumbar region was − 1.5 (range, -4.3-1.5). The most common reason for BHA was femoral neck fracture (86 patients, 75.4 %), and the most common preoperative underlying disease was hypertension (25.4 %) followed by diabetes (23.7 %) (Table 1).

**Table 1:** Patient demographic data

Variable	Values	Range
Gender, cases (%)		
Male	51 (44.7)	
Female	63 (55.3)	
Age at the time of BHA (year)	54.1 ± 8.8	29 to 59
Laterality (right/left)	55/59	
Follow-up duration (year)	13.8 ± 3.5	10 to 23
Body mass index (kg/m^2^)	22.0 ± 2.7	16.0 to 32.8
Underweight, < 18.5	5 (4.4)	
Normal weight, 18.5–24.9	88 (77.2)	
Overweight, ≥ 25	21 (18.4)	
Bone mineral density (T-score)	-1.5 ± 0.9	-4.3 to 1.5
Cause for BHA (%)		
Femoral neck fracture	86 (75.4)	
Osteonecrosis of femoral head	20 (17.6)	
Pathologic fracture	4 (3.5)	
Intertrochanteric femur fracture	4 (3.5)	
Underlying disease (%)		
Diabetes	27 (23.7)	
Dislipidaemia	2 (1.8)	
Hypertension	29 (25.4)	
Cerebrovascular accident	2 (1.8)	
Cardiac disease	4 (3.5)	
Pulmonary disease	4 (3.5)	
Hepatobiliary disease	5 (4.4)	
Kidney disease	4 (3.5)	
Cancer	5 (4.4)	
Rheumatoid arthritis	3 (2.6)	

Data are shown as mean ± standard deviation or n (%).

*BHA* bipolar hemiarthroplasty

All operations were performed by an experienced arthroplasty surgeon using a posterolateral approach with the patients in the lateral decubitus position. All patients recieved a modular bipolar femoral head (Multiploar^®^ bipolar cup; Zimmer, Warsaw, IN, USA). BHA was finally performed after confirming the cartilage status of the acetabulum during surgery. Once the femoral head was resected or removed, we measured it using a head gauge. Thereafter, we performed an endoprosthesis trial by measuring over and under 1 mm to obtain a close fit. The Fiber Metal Taper cementless stem (Versys, Zimmer) and Heritage cemented stem (Zimmer) were used in 112 and 2 hips, respectively. On the second postoperative day, the patients were instructed to walk with partial weight-bearing with the aid of crutches or walker, with full weight-bearing as tolerated.

A Postoperative radiologic review was performed at 6 weeks; 3, 6, and 12 months; and annually thereafter. Standard radiographs, with additional Judet views, were used to detect periprosthetic osteolysis and acetabular erosion. Radiolucent lesions of ≥ 2mm around the prosthetic components that were not present immediately postoperatively denoted osteolysis [12]. Acetabular erosion was graded, according to radiographic appearance, as grade 0 (no erosion), grade 1 (narrowing of the articular cartilage, no bone erosion), grade 2 (acetabular bone erosion and early migration), and grade 3 (protrusio acetabuli) [10]. The reliability of the measurements was assessed by two observers. By measuring the distance from the inter-teardrop line to the uppermost point of the lesser trochanters of the femur, we determined that difference as a degree of leg length discrepancy (LLD). LLD was expressed as a negative value for shorter than the contralateral side and a positive value for longer.

The patients were divided into two groups according to the presence of erosion in the acetabular region, and the preoperative parameters, prosthetic head size used in BHA and postoperative LLD were compared between two groups. Moreover, the risk factors related to acetabular erosion after BHA were analyzed. The medical records and radiographs of patients who underwent reoperation were analyzed to determine the cause of revision and the procedures performed.

### Statistical analysis

Summary data are expressed as means ± standard deviations for continuous variables and as number and frequencies (%) for categorical variables. Continuous variables with a non-normal distribution were analyzed using the Mann-Whitney U-test, whereas those with a normal distribution were analyzed using independent t-tests. Categorical data were statistically analyzed using the chi-square test or Fisher’s exact test (n < 40 or t < 1). The inter-observer reliability and intra-observer reproducibility of the grade of acetabular erosion were evaluated by weighted kappa with 95 % confidence intervals (CIs). Revision-free survival rate was estimated using Kaplan-Meier survival curves, with revision for any reason and acetabular erosion-related reoperation as end points. Multivariate logistic regression analysis was performed to examine the association between possible risk factors and acetabular erosion. Odds ratios (ORs) and 95 % CIs are reported for all associations. Statistical analysis was performed using SPSS software (version 20.0; SPSS Inc., Chicago, IL, USA). A *P*-value of < 0.05 was considered statistically significant.

## Results

**Fig. 2:**
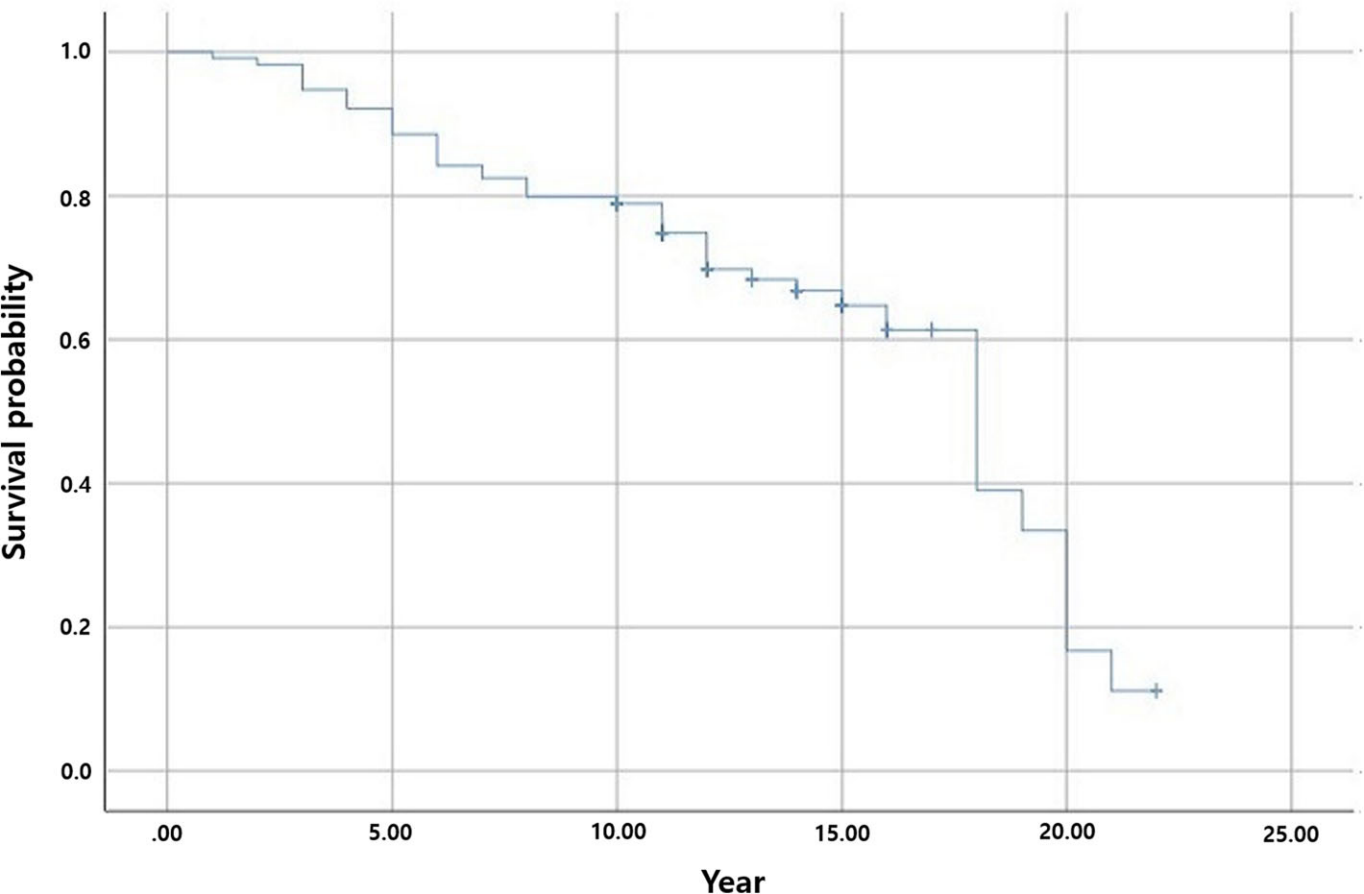
Survival analysis with all cause reoperation as the end point

**Fig. 3:**
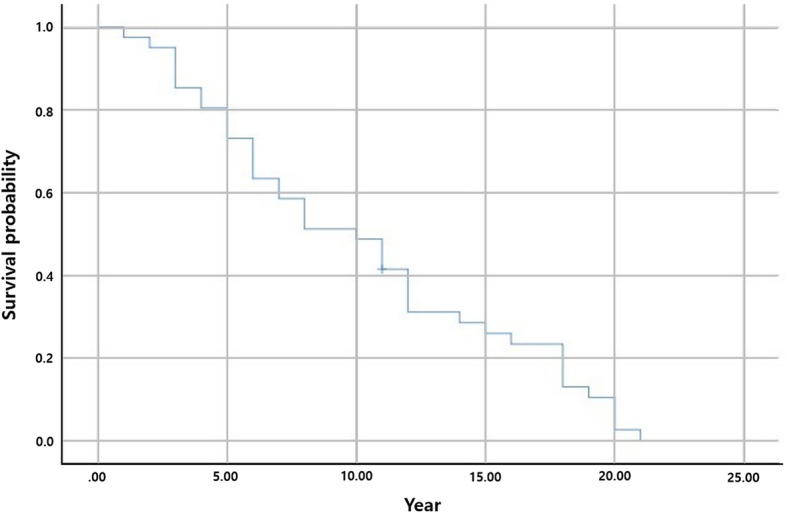
Survival analysis with acetabular erosion as the end point

At the last follow-up, reoperation was performed in 44 of the 114 patients (38.6 %). The most common reason for reoperation was acetabular erosion and/or destruction in 39 patients (88.6 %), and other reasons included stem loosening, periprosthetic femoral fracture, and recurrent instability (Table 2). When reoperation was needed because of acetabular erosion, conversion to THA was performed in all cases. Regardless of the acetabular erosion grade, reoperation was performed when the patient wanted reoperation due to pain. The distribution of the grade of acetabular erosion according to Baker’s classification at the final follow-up images was grade 0 in 73 patients (64 %), grade 1 in 24 patients, grade 2 in 7 patients, and grade 3 in 10 patients. The inter-observer reliability and intra-observer reproducibility of the grade of acetabular erosion were excellent with a high weighted kappa value (> 0.7). When all-cause reoperation was the end point using the Kaplan-Meier survival curve, the survival rate reduced time dependently: 88.6 % at 5 years, 78.9 % at 10 years, and 64.7 % at 15 years after surgery (Fig. 2). When reoperation related to acetabular erosion was the end point, the survival rate was significantly more time dependent: 73.2 % at 5 years, 48.8 % at 10 years, and 25.9 % at 15 years (Fig. 3).

**Table 2:** Implant survival and grade of acetabular erosion

			Number (%)			
Implant	Maintain		70 (61.4)			
	Reoperation		44 (38.6)			
	Acetabular erosion		39 (88.6)			
	Stem loosening		2 (4.5)			
	Periprosthetic fracture		2 (4.5)			
	Instability		1 (2.3)			
					Weighted kappa (95 % CIs)	
					Intra-observer	Inter-observer
Grade of acetabular erosion	Grade 0		73 (64.0)		0.781 (0.743 to 0.833)	0.700 (0.656 to 0.762)
	Grade 1		24 (21.1)		0.732 (0.683 to 0.797)	0.721 (0.661 to 0.783)
	Grade 2		7 (6.2)		0.721 (0.669 to 0.759)	0.714 (0.658 to 0.769)
	Grade 3		10 (8.7)		0.783 (0.750 to 0.847)	0.733 (0.685 to 0.801)
Total			114			

*CI* confidence interval

The patients were categorized into two groups according to the presence of acetabular erosion: 69 patients with no change in the acetabular side (group 1) and 45 patients with acetabular erosion (group 2). The comparison of preoperative demographics between the two groups showed that the proportion of men in group 2 was high (*p* = 0.024) and the average age was 47.9 years, which was statistically significantly young (*p* < 0.001). The mean preoperative BMI was 24.0 kg/m^2^ in group 2, which is higher than that in group 1 (*p* < 0.001). With respect to the disease that necessitated BHA, femoral neck fracture was more frequent in group 1 (*p* < 0.001) and avascular necrosis of the femoral head was more frequent in group 2 (*p* < 0.001). Hypertension and pulmonary disease were more common in group 2 (*p* = 0.045 and *p* = 0.022, respectively). The mean head size of the BHA prosthesis used in surgery was 46.0 mm in group 1 (no acetabular erosion) was smaller than that used in group 2 (*p* < 0.001). No differences between the two groups were found with respect to preoperative BMD, other underlying diseases and postoperative LLD (Table 3).

**Table 3:** Univariate and multivariate analysis to verify the correlation and predictive power for a higher risk of acetabular erosion after BHA

Variable	No erosion (*N* = 69)	Erosion (*N* = 45)	*p* value	Univariate		Multivariate	
				OR (95 % CIs)	*p* value	OR (95 % CIs)	*p* value
Age (year)	58.1 ± 2.4	47.9 ± 11.2	< 0.001	0.774 (0.687 to 0.871)	< 0.001	0.787 (0.631 to 0.981)	0.033
Gender (male/female)	22/47	29/16	0.024	2.408 (1.117 to 5.195)	0.025	0.851 (0.149 to 4.859)	0.856
BMI (kg/m^2^)	20.7 ± 1.3	24.0 ± 3.1	< 0.001	2.400 (1.684 to 3.421)	< 0.001	1.903 (1.331 to 2.722)	< 0.001
<18.5	4	1					
18.-24.9	66	22					
≥25	3	18					
BMD (T-score)	-1.5 ± 0.5	-1.5 ± 1.4	0.781	1.070 (0.781 to 1.593)	0.741		
Cause for BHA							
FNF	62	24	< 0.001	7.750 (2.919 to 20.579)	< 0.001	4.390 (0.161 to 11.558)	0.380
ONFH	1	19	< 0.001	9.692 (6.327 to 39.290)	< 0.001	15.093 (0.237 to 96.508)	0.200
PathF	4	0	0.152	N/A	0.999		
ITF	2	2	0.647	1.558 (0.221 to 11.479)	0.663		
Underlying disease							
Diabetes	12	15	0.050	2.375 (0.987 to 5.717)	0.054		
Dislipidaemia	0	2	0.154	N/A	0.999		
Hypertension	13	16	0.045	2.377 (1.007 to 5.607)	0.048	2.950 (0.676 to 12.870)	0.150
CVA	0	1	0.395	N/A	1.000		
Cardiac Dz	1	3	0.299	4.875 (0.489 to 48.235)	0.177		
Pulmonary Dz	0	4	0.022	N/A	0.999		
Hepatobiliary Dz	0	2	0.154	N/A	0.999		
Kidney Dz	2	1	1.000	1.313 (0.116 to 14.925)	0.826		
Cancer	3	2	1.000	1.023 (0.164 to 6.379)	0.980		
RA	0	3	0.059	N/A	0.999		
Head size (mm)	46.0 ± 3.1	48.2 ± 3.1	< 0.001	1.258 (1.102 to 1.436)	0.001	1.153 (0.889 to 1.494)	0.282
Postoperative LLD (mm)	0.5 ± 2.7	0.6 ± 1.5	0.656	0.914 (0.887 to 1.015)	0.798		

Data are shown as mean ± standard diviation or n

*OR* odds ratio; *CI* confidence interval; *BHA* bipolar hemiarthroplasty; *BMI* body mass index; *BMD* bone mineral density*; FNF* femoral neck fracture; *ONFH* osteonecrosis of the femoral head; *PathF* pathologic fracture; *N/A* not available; *ITF* intertrochanteric femur fracture; *CVA* cerebrovascular accident; *Dz* disease; *RA* rheumatoid arthritis; *LLD* leg length discrepancy

Young age, male sex, high BMI, avascular necrosis of the femoral head, hypertension, and small head size of the BHA prosthesis were statistically associated with acetabular erosion in the univariate model and were entered into multiple logistic regression analysis for the risk factors of the acetabular erosion after BHA. The final multivariate logistic regression analysis, after adjustment for other risk factors, showed that young age at the time of surgery (OR 0.787, 95 % CI 0.631–0.981, *p* = 0.033) and high BMI (OR 1.903, 95 % CI 1.331–2.722, *p* < 0.001) were independent risk factors for acetabular erosion after BHA in patients aged < 60 years. Other variables such as sex, preoperative BMD, underlying disease, and postoperative LLD were not associated with the occurrence of acetabular erosion after BHA.

## Discussion

In this retrospective cohort study, acetabular erosion occurred at a relatively high frequency when BHA was performed in young patients, increasing the conversion rate to THA. This demonstrated that acetabular erosion after BHA was highly related to age at the time of surgery and BMI, which was closely related to the patients’ activity but had little relationship to other factors including poor bone quality. The strengths of this study were the analysis of relatively large follow-up data from young patients and the study design in which prostheses from a single manufacture were implanted in consecutive patients by one surgeon.

Many reports have presented a comparison of the results of BHA and THA [3, 13, 14]. BHA has an advantage in terms of preventing postoperative dislocation and is considered a good option when the acetabular cartilage is preserved in old-aged patients [1–3, 8, 9]. Moreover, BHA is still used as a salvage procedure for patients with poor general condition or tumors [15, 16]. In the case of THA, despite its excellent postoperative functional outcomes, its use is limited in patients with old age or musculoskeletal disorders due to dislocation-related problems. In recent years, the frequency of use of BHA had gradually decreased because of the wear resistance of a polyethylene liners, increased use of ceramic bearing, and dual mobility in THA. However, BHA is still required in some cases, determination of long-term survival after BHA requires reports of the long-term results. However, long-term follow-up is very difficult owing to low life expectancy and low compliance in old-aged patients. For these reasons, we targeted young patients who are relatively easy to follow-up. Further, the association between acetabular erosion and the patients’ activity could be analyzed by targeting younger patients with higher activity than older patients.

Several reports have been published on the risk factors associated with acetabular erosion after BHA. Kwok et al. surmised that leaving a longer neck may cause overtightening of the periprosthetic soft tissues leading to increased stress across the hip joint, and resultant increased wear [17]. However, their data were limited owing to the small number of patients, and, in reality, the goal of hip arthroplasty is to prevent leg length discrepancy. In elderly patients, the soft tissue tension during surgery is weak and surgery is often performed for a longer leg length. Unlike these reports, there was no statistical difference in postoperative LLD between the two groups, and no association with the occurrence of acetabular erosion after BH was found in this study. At present, rather than increasing the leg length, it is necessary to re-check the offset or evaluate and correct impingement or the soft tissue condition.

Other studies suggested that a smaller femoral head size is another risk factor associated with acetabular erosion after BHA. Schiavi et al. reported that a smaller head size of the BHA prosthesis leads to polar wear, implying a higher risk of acetabular erosion and migration. In their population, this risk was consistent with the use of an implant head < 48 mm in diameter [18]. A small head distributes all forces to a rather small area of articular cartilage within the acetabulum, whereas a larger head transmits all forces initially at the entrance to the acetabulum [19]. However, the femoral head size when performing BHA is actually the patient’s own measurement. In other words, a small or large femoral head size is only the operator’s relative choice for the patient’s native size. Therefore, the result according to the absolute size of the femoral head is important and no difference was noted in this study. That is, if the femoral head is accurately measured using a template before surgery and a calibre during surgery, it is reasonable to presume that it has no effect, as the present results showed.

In a study of 69 patients who underwent Thompson hemiarthroplasty, Phillips reported that the physical activity level and duration of follow-up had the highest correlation with the severity of acetabular erosion [20]. Moreover, obesity was also reported as a risk factor for acetabular erosion [21]. Presumably, increased body weight leads to increased wear of the acetabulum, causing more acetabular erosion. The present results showed that acetabular erosion after BHA was highly related to age at the time of surgery and BMI, which is closely related to the patients’ physical activity. However, sex, preoperative bone quality, and underlying disease were not associated with the occurrence of acetabular erosion after BHA. Studies on avascular necrosis of the femoral head demonstrated that degenerative changes of the acetabular cartilage are common in patients with osteonecrosis of the femoral head, even when radiographs of the acetabulum appear normal [22, 23]. In such cases, evidence showed that BHA resulted in unacceptably high failure rates, mainly owing to central migration of the prosthetic femoral head. In the multiple logistic regression analysis for the risk factors of acetabular erosion after BHA in this study, avascular necrosis of femoral head, which was statistically associated with acetabular erosion in the univariate model, was not an independent risk factor in the final multivariate logistic regression.

Among 44 reoperations after BHA, reoperation was performed in five hips for a cause other than acetabular problems, including stem loosening, periprosthetic femoral fracture, and recurrent instability. In this study, the incidence of acetabular erosion after BHA continued to increase during the follow-up, and reoperation was performed at a mean of 10.2 years after surgery. In other words, it is difficult to expect long-term safety with BHA when considering reoperation for acetabular erosion in patients with an expected survival of > 10 years, or in those with good physical activity levels.

This study had some limitations. First, this was a single-center retrospective cohort study. However, we accounted for all postoperative radiologic outcomes in our consecutive patients. Second, although most of the patients had a femoral neck fracture, surgery was needed for various diseases. The possibility that the disease type requiring surgery affects acetabular erosion after BHA cannot be excluded. Third, a comparative analysis according to the femoral stem fixation method (i.e. cemented vs. cementless) was not performed. Cemented stems have the potential advantage of a reduced risk of periprosthetic fracture in the elderly population with poor bone integrity. However, cemented stems also may carry the risk of increased operative time and perioperative mortality secondary to fat and bone marrow emboli when compared to cementless stems. In this study, we focused on the follow-up results for more than 10 years related to acetabular erosion after BHA. Finally, 25 % of our patients who underwent initial BHA were incompletely followed-up. There was a risk of patents going to other hospitals for their reoperations in these patients. Although patient compliance to clinical follow-up after BHA remains challenging, the 114 patients followed up for > 10 years do not represent a small number. These limitations are obvious obstacles to the generalization of our results, and further multicenter prospective studies are needed to verify their authenticity. We are also continuing further follow-up in these patients.

## Conclusions

Our minimum 10-year follow-up outcomes of BHA in patients aged < 60 years showed a relatively high conversion rate to THA. In particular, age and BMI at the time of surgery were identified as independent risk factors for acetabular erosion after BHA. When considering BHA in younger patients, more careful decisions should be made with respect to patient’s choice, keeping in mind that long-term survival cannot be guaranteed. In other words, in relatively young patients with high activity, especially those with high BMI, the use of BHA should be carefully determined and THA should be considered for long-term prosthesis survival. Moreover, because of the risk of more frequent reoperations in the future, this patient population should be continuously monitored to evaluate the longevity of BHA.

## Data Availability

The data utilized are accessible from the corresponding author upon reasonable request.

## Abbreviations

BHA: Bipolar hemiarthroplasty
BMD: Bone mineral density
BMI: Body mass index
CIs: Confidence intervals
ORs: Odds ratios
THA: Total hip arthroplasty
